# Polyhydroxybutyrate production by freshwater SAR11 (LD12)

**DOI:** 10.1093/ismejo/wraf087

**Published:** 2025-04-30

**Authors:** Brittany D Bennett, David A O Meier, V Celeste Lanclos, Hasti Asrari, John D Coates, J Cameron Thrash

**Affiliations:** Department of Biological Sciences, University of Southern California, Los Angeles, CA 90089, United States; Department of Plant and Microbial Biology, University of California, Berkeley, Berkeley, CA 94720, United States; Department of Biological Sciences, University of Southern California, Los Angeles, CA 90089, United States; Department of Biological Sciences, University of Southern California, Los Angeles, CA 90089, United States; Department of Plant and Microbial Biology, University of California, Berkeley, Berkeley, CA 94720, United States; Department of Biological Sciences, University of Southern California, Los Angeles, CA 90089, United States

**Keywords:** SAR11, LD12, polyhydroxybutyrate, microbial ecology, microbial physiology

## Abstract

SAR11 bacteria (order *Pelagibacterales*) are oligotrophs and often the most abundant bacterioplankton in aquatic environments. A subset of sequenced SAR11 genomes, predominantly in the brackish and freshwater SAR11 subclades, contain homologs of *pha* genes, which in other organisms confer the ability to store carbon and energy via polyhydroxyalkanoate (PHA) polymers. Here, we investigated the relevance of PHA production to SAR11 biology. Phylogenetics showed that Pha proteins occurred on a long branch and provided evidence for origin at the common ancestor of the brackish IIIa and freshwater LD12 subclades, followed by horizontal transfer within SAR11. Using the LD12 representative “*Candidatus* Fonsibacter ubiquis” strain LSUCC0530, we found that many LSUCC0530 cells contained a single Nile red-staining granule, confirmed that the cells produced polyhydroxybutyrate, a common form of PHA, and estimated the total polyhydroxybutyrate content in the cells. We heterologously expressed the LSUCC0530 *phaCAB* locus in *Escherichia coli*, finding it to be functional and the likely origin of the polyhydroxybutyrate. We also determined that, irrespective of changes to carbon, nitrogen, and phosphorus concentrations, a similar fraction of LSUCC0530 cells generated polyhydroxybutyrate granules and expression of the *phaCAB* locus remained constant. We suggest that polyhydroxybutyrate synthesis in LSUCC0530 may be constitutively active due to the slow growth dynamics and minimal regulation that characterize SAR11 bacteria. This work characterizes polymer storage in SAR11, providing new insights into the likely fitness advantage for cells harboring this metabolism.

## Introduction

Alphaproteobacterial strains within the order *Pelagibacterales*, colloquially the SAR11 clade, are ubiquitous, highly abundant, slow-growing members of the bacterioplankton in aquatic ecosystems ranging from marine to freshwater [1–4]. Although the majority of research on SAR11 has focused on marine oligotrophs that frequently dominate nutrient-poor offshore surface communities [3, 5, 6], including the well-described SAR11 subclade Ia strain “*Candidatus* Pelagibacter ubique” HTCC1062 [2], SAR11 also comprises groups that are adapted for brackish (subclade IIIa) [7] and fresh water (subclade IIIb, also called LD12) [8, 9], which often make up the largest fraction of low-salinity bacterioplankton [10, 11].

Regardless of environmental habitat, all SAR11 bacteria have highly streamlined genomes with limited metabolic pathways and few annotated regulatory genes [9, 12–14], resulting in a high degree of constitutive gene expression [13, 15, 16], making them both particularly fit for low-nutrient conditions and less flexible in response to changes in their environmental conditions [4, 9, 12, 13, 17]. SAR11 genomes also share large percentages of their genes even across subclades, resulting in similar overall metabolic profiles despite hundreds of millions of years of divergent evolution [9, 18]. Nevertheless, a distinguishing genomic feature that may provide a means of niche adaptation in some SAR11 lineages is the potential for carbon polymer storage [7, 19, 20]. Homologs of polyhydroxyalkanoate (PHA) synthesis genes occur in multiple SAR11 isolate and metagenome-assembled genomes from both brackish and freshwater environments, predominantly in the IIIa and LD12 subclades [7, 19, 20]. However, the function and possible fitness benefit this pathway might confer on the few SAR11 groups that harbor it still need investigation.

PHA production is a carbon and energy storage mechanism widespread throughout the Bacteria [21]. In fact, some PHA-producing microbes can store up to ~70% of their dry mass as PHA [22–24]. Depending on the *pha* genes present in an organism’s genome and the available carbon source(s), PHA polymers composed of monomers with varying side chain lengths and structures may be produced [25], though the most well-studied PHA is polyhydroxybutyrate (PHB). A common PHA production pathway requires three core enzymes: acetyl-CoA acetyltransferase PhaA (in some organisms designated PhbA), acetoacetyl-CoA reductase PhaB (or PhbB), and PHA synthase PhaC (or PhbC) [26, 27]. Together these enzymes form a three-step pathway in which (in organisms producing PHB) two acetyl-CoA molecules are synthesized into acetoacetyl-CoA, which is reduced to the monomer unit 3-hydroxybutyryl-CoA, and then these monomers are polymerized into PHB (illustrated in [25]). Dozens of alternative monomer units, such as 3-hydroxyvalerate, 3-hydroxyoctanoate, and 3-hydroxydecanoate, have been found incorporated into PHA polymers [28]. Both the available metabolites and the substrate specificity of an organism’s PhaC determine which monomer(s) may be used (reviewed in [29].

Regulation of PHA production is multilayered and can occur at the transcriptional, translational, and/or post-translational levels, though the mechanisms vary by organism (reviewed in [30, 31]). Most regulatory mechanisms discovered thus far appear to affect the amount of PHA accumulated in the cell, whereas the monomer composition is usually determined by the carbon source(s) provided to the organism [32]. Generally, a combination of nutrient limitation, particularly of nitrogen and/or phosphate, and carbon abundance will induce PHA storage in organisms that have this capacity [33, 34]. Carbon may be shunted toward PHA formation when other nutrients have been exhausted due to allosteric regulation of PhaC and/or PhaA by metabolic pathway intermediates [35–37]. Additionally, in various organisms there is a range of upstream mechanisms that regulate the *pha* locus in response to environmental conditions (reviewed in [31]). Given the low number of regulatory elements encoded in SAR11 genomes and their oligotrophic adaptations, it was unclear to us what, if any, regulatory mechanism(s) governing PHA production might exist in this clade. Furthermore, the type of PHA produced and the amount stored per cell will aid in understanding how SAR11 organisms use this metabolism.

In this work, we present evidence that carbon storage in the form of PHA polymers may confer a fitness benefit to the cultured LD12 representative “*Candidatus* Fonsibacter ubiquis” strain LSUCC0530. Previously, a putative *pha* locus was identified in the LSUCC0530 genome [7]. Here, we use genetic, phenotypic, and chemical analyses to demonstrate that this locus is functional and that LSUCC0530 produces PHA granules under all tested growth conditions. We postulate that LSUCC0530 synthesizes PHA regardless of environmental nutrient levels, and that this stored carbon may aid the bacterium during starvation periods. Furthermore, the phylogenetic similarity of LD12 PHA production genes to those in other SAR11 strains suggests a common strategy that may have been horizontally transferred within the clade.

## Materials and methods

### Bacterial strains and growth conditions

Bacterial strains and plasmids used in this study are listed in Table 1. We grew LSUCC0530 in polycarbonate flasks at room temperature (~19–21°C) in either JW5 (Table S1) [38] or CCM5PK (Table S1), and HTCC1062 in polycarbonate flasks at room temperature in AMS1 supplemented with 10 μM L-methionine, 50 μM L-glycine, and 50 μM sodium pyruvate (Table S1) [39]. For conditions lacking a supplemental carbon source, a carbon-depleted version of JW5 (Table S1) was used for LSUCC0530, and a version of AMS1 lacking sodium pyruvate was used for HTCC1062. SAR11 strains were grown in the dark and without shaking unless otherwise noted. We grew *Escherichia coli* at 37°C in Lennox Luria Broth (Sigma-Aldrich, St. Louis, MO) supplemented with 50 μg/ml kanamycin (Sigma-Aldrich), 300 μM diaminopimelic acid (Sigma-Aldrich), and/or 200 mM isopropyl ß-D-1-thiogalactopyranoside (AmBeed, Arlington Heights, IL) where applicable; liquid cultures were shaken at 180 rpm. We grew *Cupriavidus necator* H16 at 30°C in nutrient broth (BD Difco, Franklin Lakes, NJ) supplemented with 0.2% (wt/vol) sodium gluconate when necessary to induce PHA production; liquid cultures were shaken at 180 rpm.

**Table 1:** Bacterial strains and plasmids used in this study.

**Strain**	**Description**	**Reference**
LSUCC0530	*``Candidatus* Fonsibacter ubiquis,'' SAR11 subclade IIIb (LD12) type strain	Henson et al. 2020 ISMEJ
HTCC1062	*``Candidatus* Pelagibacter ubique,'' SAR11 subclade Ia type strain	Rappe et al. 2002 Nature
UQ950	*Escherichia coli* DH5α λ(pir) cloning host; F-Δ(a*rgF*-*lac*)169 (5) *Φ80dlacZ58*(ΔM15) *glnV44*(amber (UAG) suppressor) *rfbD1 gyrA96*(NalR) *recA1 endA1 spoT1 thi-1 hsdR17 deoR* λ*pir*+	Saltikov et al. 2003 PNAS
WM3064	*E. coli* conjugation strain; *thrB1004 pro thi rpsL hsdS lacZ*ΔM15 RP4–1360 Δ(*araBAD*)567 Δ*dapA1341*::[*erm pir*(wild type)]	Saltikov et al. 2003 PNAS
MG1655	*E. coli* K-12, wild type; F- lambda- *ilvG- rfb*-50 *rph*-1	Blattner et al. 1997 Science; Provided by Steven Finkel
BDB256	MG1655 with empty pBBR1MCS-2	This work
BDB253	MG1655 with pBBR1MCS-2::*phaCAB_CN_*	This work
BDB257	MG1655 with pBBR1MCS-2::*phaCBA_FU_*	This work
H16	*Cupriavidus necator* (ATCC 17699)	Purchased from American Type Culture Collection (Manassas, VA)
**Plasmid**	**Description**	**Source**
pBBR1MCS-2	Broad-host-range cloning vector, Km^r^	Kovach et al. 1995 Gene
pBBR1MCS-2::phaCAB*_CN_*	H16_A1437–A1439, 21 bp upstream, 4 bp downstream; Km^r^	This work
pBBR1MCS-2::*phaCBA_FU_*	Ga0258990_1170, 20 bp upstream, 3 bp downstream; Ga0258990_1172–1173, 3 bp upstream, 45 bp downstream; Km^r^	This work

### Growth curves

We inoculated cryostocks of SAR11 strains into triplicate 25 ml starter cultures of either JW5 (LSUCC0530) or AMS1 (HTCC1062) and grew them to late exponential phase. We generated growth curves by inoculating each starter culture to a cell density of ~2500 cells/ml into 25 ml of either JW5 (LSUCC0530) or AMS1 (HTCC1062), with or without organic carbon sources as noted. We monitored growth by periodically staining aliquots with 1x SYBR green I (Lonza, Basel) and enumerating them on an Accuri C6 Plus flow cytometer (BD Biosciences) (ex 488 nm, em 533/30). Each growth curve was performed with biological triplicates at least twice. We calculated growth rates in Prism 5 by first fitting a nonlinear regression (Y=Y_0_*exp(k*X), where Y = cells/ml, X = time, and k = rate constant) to the exponential (linear) phase of the growth curve, then calculating doubling time = ln [2]/k.

### Transmission electron microscopy

We grew LSUCC0530 cells in JW5 to late exponential phase, processed them as described previously [7], and took images on a JEM-1400 transmission electron microscope. PHB content was estimated by measuring the volume of each inclusion body in Fiji ver. 2.15.0 running ImageJ ver. 1.54f [40] and multiplying by the density of amorphous PHB [41].

### Fluorescence microscopy

We prepared LSUCC0530 cultures as follows: 2 ml of culture was centrifuged for 30 min at 17500 × g. The top 1.92 ml of supernatant was carefully pipetted off and discarded, and 500 μl 35% EtOH in PBS (per L: 8 g NaCl, 201 mg KCl, 1.42 g Na2HPO4, 245 mg KH2PO4, pH 7.4) was added to the cell pellet. Cells were vortexed vigorously and incubated 15 min at room temperature. Cells were centrifuged again for 30 min at 17500 × g, after which the top 500 ul of supernatant was carefully pipetted off and discarded. We added 200 μl PBS to each cell pellet and vortexed pellets vigorously. We added 0.6 μl of 1 mg/ml DAPI (Thermo Scientific, Waltham, MA) and 0.6 μl of 10 mg/ml Nile red (Sigma-Aldrich) in 100% DMSO to the cell suspension, mixed cells gently by inversion, and incubated them in the dark for 30 min. We mounted 6 μl of stained cells onto a slide affixed with a 1% agarose pad [42], topped with a #1 coverslip followed by a drop of Type B immersion oil (Cargille, Cedar Grove, NJ), and imaged on an Eclipse Ti epifluorescence microscope (Nikon Instruments, Melville, NY) equipped with a Plan Apo 100x/1.40 oil immersion objective, X-Cite 110LED light source (Excelitas Technologies, Waltham, MA), and DAPI (ex 360–380 nm, em 435–485 nm) and red-shifted TRITC bandpass (ex 528–553, em 590–650) filter cubes. Rates of Nile red-positive cells were calculated from all cells observed within five fields of view for each sample; data for each growth condition represent aggregated results of six experiments, each consisting of three biological triplicates.

We prepared *Escherichia coli* cultures as follows: 100 μl of liquid overnight culture was centrifuged for 1 min at 8000 × g. The supernatant was poured off and discarded, and the cell pellet was resuspended in 500 μl PBS and then centrifuged for 1 min at 8000 × g. The supernatant was discarded, and cells were resuspended in 500 μl 35% EtOH in PBS and then incubated at room temperature for 15 min. Cells were centrifuged for 1 min at 8000 × g, and the supernatant was discarded. The cell pellet was resuspended in 500 μl PBS, and 0.5 μl of 1 mg/ml DAPI and 1.5 μl of 1 mg/ml Nile red in 100% DMSO were added. Cells were mixed gently by inversion and incubated in the dark for 30 min before mounting onto slides and imaging as above. Each *E. coli* strain was imaged on three separate occasions.

### Phylogeny of Pha proteins

We queried the PhaC, PhaA, and PhaB protein sequences from LSUCC0530 and the PhaC1, PhaA, and PhaB1 protein sequences from *C. necator* H16 twice in PSI-BLAST [43] against the RefSeq Select database: once against the full database, and once limiting the search to the SAR11 cluster, using as many iterations as necessary to reach convergence. We selected the top 250 proteins returned for each search, minus “Multispecies” hits, for inclusion in the analysis. To fill in the SAR11 section of the tree and represent a broad range of strains, collection sites, and query coverages, we also queried LSUCC0530 Pha protein sequences in PSI-BLAST against the nr database, once against the full database and once limiting the search to the SAR11 cluster. We hand-picked the following sequences from this search: 17 additional SAR11 and one *Rickettsiales* PhaC homologs, 33 additional SAR11 and nine other *Alphaproteobacteria* species PhaA homologs, and 15 additional SAR11 PhaB homologs. We also manually added a further 14–18 homologs previously identified in a comparative genomic analysis of the SAR11 clade [7] to each protein family. All query results for each enzyme were combined into one sequence file and de-duplicated. Sequences were aligned with MUSCLE v3.8.1551 [44] and trimmed with trimAl v1.4.1 [45] using default settings. Phylogenetic trees were inferred with IQ-TREE v2.0.6 [46] using 1000 ultrafast bootstrap approximation replicates and model LG + R8 (PhaA), LG + R7 (PhaB), or LG + R9 (PhaC); visualization was performed in iTOL v6 [47].

### Plasmid construction

Primers used to construct the expression vectors in this study are listed in Table 2. Expression of *phaCBA_FU_* was achieved by ligating *phaC* (Ga0258990_1170) to *phaBA* (Ga0258990_1172–1173) cloned from the LSUCC0530 genome and inserting the fusion into the multiple cloning site of pBBR1MCS-2 [48]. Expression of *phaCAB_CN_* was achieved by inserting *phaC1AB1* (H16_A1437–A1439) cloned from the *C. necator* H16 genome into the multiple cloning site of pBBR1MCS-2.

**Table 2:** Primers used in this study.

**Primer**	**Sequence**	**Restriction Site**
pha *gene insertion into pBBR1MCS-2*		
H16phaCF	CTAGgaattcCGGCAGAGAGACAATCAAATC	EcoRI
H16phaBR	CTAGggatccCAGGTCAGCCCATATGCAGG	BamHI
530phaCF	CTAGggatccGCTATTATCAAAATCATAAAATGAC	BamHI
530phaCR	CATGggtaccCAATCATGACTGTTTTACGTATTG	KpnI
530phaBF	CATGggtaccCAAATGTCTAAAGTTGCACTAG	KpnI
530phaAR	CTAGgagctcGGCTTGCTCAGCTCTTAC	SacI
*Expression analysis*		
530phaCexpF	CTCAGACTCCACTCGTCTTCCTG	
530 phaCexpR	GGAGCGATATGGTCTTCAGTTGTTG	
530phaBAexpF	ACTTGCTTCCGTTGCCTTTGG	
530phaBAexpR	TGTCACCCAACTTCACACCATTTC	
dnaXexpF	GATTTACCAAGCCCAGAGCAAC	
dnaXexpR	GGAGTTCGATCAATTTCCTCTGTC	

### Lipid extraction and GC/MS

We collected LSUCC0530 cells grown in 2 liters of CCM5PK or *C. necator* H16 cells grown in 50 ml of nutrient broth supplemented with sodium gluconate by vacuum filtration onto 25-mm diameter, 0.1-μm pore polyvinylidene fluoride membrane filters (Durapore, Darmstadt), which were placed in a 50-ml conical polypropylene tube and frozen at −80°C. We extracted lipids from cells on filters and performed GC/MS similarly to a previously described protocol [49]. Briefly, we added 1.5 ml chloroform and 1.5 ml acidified methanol to glass culture tubes containing the filters (enough to fully submerge the filters in solvent), capped the tubes with screw-tops containing PTFE/silicone septa, and vortexed for 1 min. Tubes were incubated for 2.5 h at 100°C on a heating block, then cooled on ice for 5 min. We washed samples by adding 500 μl double-deionized H2O and vortexing briefly. We removed the bottom, organic layer into 150 μl inserts in GC glass vials and capped these with PTFE/silicone septa. Samples were analyzed by GC/MS on an 7890A GC system (Agilent Technologies, Santa Clara, CA) equipped with a DB-WAX UI column as stationary phase coupled with an Agilent 5975C inert XL EI/CI Mass Selective Detector with Triple-Axis Detector. The GC temperature gradient was programmed as follows: initialization, 80°C for 2 min; ramp 1, to 210°C at 10°C/min; ramp 2, to 250°C at 50°C/min, hold 1 min. We used poly((R)-3-hydroxybutyrate-co-(R)-3-hydroxyvalerate-co-(R)-3-hydroxyhexanoate) (Sigma-Aldrich, USA) to generate the standard curve. GC/MS experiments were performed on two independent sets of cultures.

### Reverse transcription and quantitative PCR

We extracted RNA from biological triplicates of LSUCC0530 cultures as follows: Cells of LSUCC0530 grown in 1 liter of JW5 with 1x (5 μM) or 10x (50 μM) ammonium (NH_4_Cl) and 1x (6.5 μM) or 10x (65 μM) phosphate (KH_2_PO_4_), as noted, were collected onto 25-mm, 0.1-μm pore polyethersulfone membrane filters. We added 2 ml RNAprotect Bacteria Reagent (Qiagen, Hilden) to each filter and incubated for 15 min at room temperature before vacuuming through the filter. Each filter was placed in a 2-ml microcentrifuge tube, to which 300 μl lysozyme (15 mg/ml in10 mM Tris-Cl and 1 mM EDTA) was added, and vortexed for 10 s; filters were then shaken for 5 min to lyse cells. We added 1.5 ml of Buffer RLT Plus (Qiagen) containing 15 μl β-mercaptoethanol and 1.125 μl Reagent DX (Qiagen) and vortexed the tubes vigorously. RNA was extracted from each filter with an AllPrep kit (Qiagen), including treatment with RNase-Free DNase (Qiagen) per manufacturer protocol. Extracted RNA was treated with 2 μl DNase RQ1 (Promega) for 60 min to remove any remaining contaminating DNA.

We synthesized cDNA from RNA using Luna RT Mix (New England Biolabs, Ipswich, MA) and measured gene expression by qPCR performed with 2X Universal SYBR Green Fast qPCR Mix (ABclonal, Woburn, MA) under the following conditions: 95°C for 3 min; 45 cycles of 95°C for 5 s, 57°C for 10 s, 60°C for 35 s; and melting curve acquisition from 60°C to 95°C. We confirmed using serial 10-fold dilutions of PCR-amplified target DNA that the qPCR primer pairs (listed in Table 2) had efficiencies between 90 and 110% under these conditions. Cycle thresholds (Ct) for each sample were normalized to those of the reference gene *dnaX* (ΔCt = target gene Ct – *dnaX* Ct); ΔΔCt values were calculated by subtracting the average ΔCt for the 1x JW5 cultures; fold changes were calculated as 2^(−ΔΔCt). RNA extraction and RT-qPCR analysis were performed on two independent sets of cultures, with biological triplicates for each condition.

**Figure 1:**
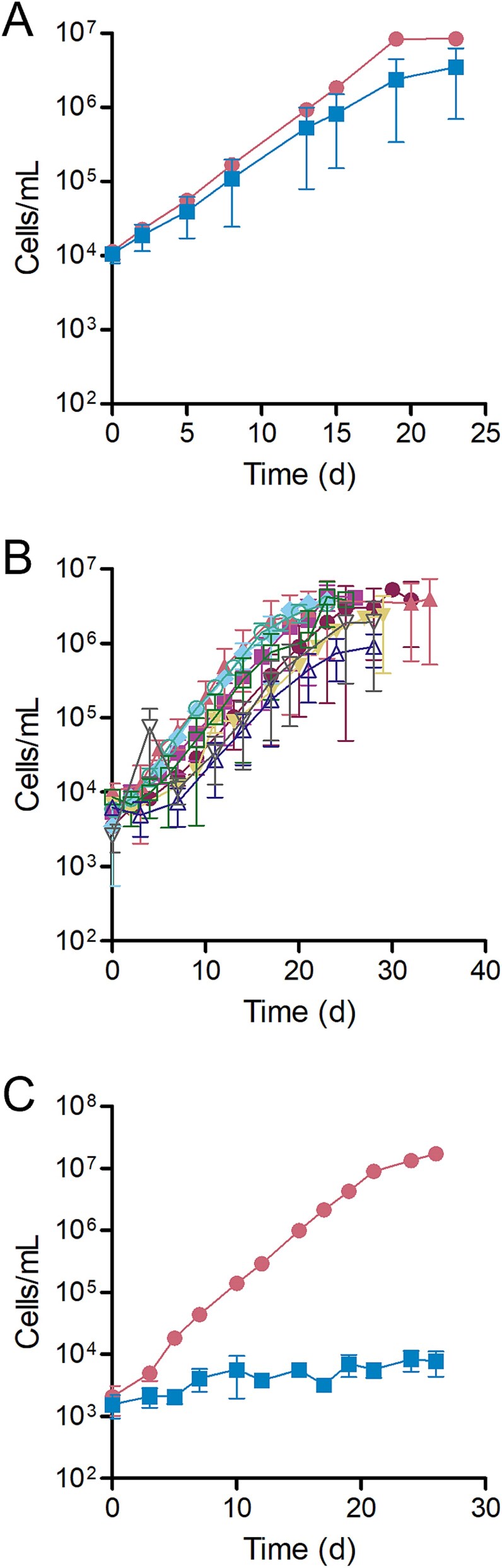
**LSUCC0530 grows for many generations without a supplemental carbon source.** (A) The rate of growth was measured for LSUCC0530 grown in JW5 with (circles) and without (squares) supplemental carbon. (B) The rate of growth was measured for LSUCC0530 grown in JW5 without supplemental carbon over nine serial transfers (T1–9); closed circles, T1; closed squared; T2, closed triangles, T3; closed inverted triangles, T4; diamonds, T5; open circles, T6; open squares, T7; open triangles, T8; open inverted triangles, T9. (C) The rate of growth was measured for “Ca”. Pelagibacter ubique HTCC1062 grown in AMS1 with (circles) and without (squares) supplemental pyruvate. Results represent the means from three biological replicates ±1 standard deviation.

## Results

### “*Ca*. F. ubiquis” replicates without a supplemental carbon source

We observed that when transferred from JW5, a carbon-replete medium, into a version of JW5 lacking supplemental carbon sources (Table S1), LSUCC0530 grew at nearly the same rate (minimum doubling time 2.19 ± 0.27 d and 2.02 ± 0.01 d, respectively) and approached a similar cell density (3.5x10^6^ ± 2.8x10^6^ cells/ml and 8.3x10^6^ ± 1.3x10^6^ cells/ml, respectively) as when transferred into fresh carbon-replete JW5 (Fig. 1A). Indeed, when we serially transferred LSUCC0530 into new cultures lacking supplemental carbon, the cells continued to grow through up to nine transfers (Fig. 1B; Fig. S3), representing over 80 generations. This ability of LSUCC0530 to grow without a carbon source is quite different from other well-studied SAR11 strains such as “*Ca*. Pelagibacter ubique” HTCC1062, which was unable to grow upon inoculation into medium without supplemental carbon (Fig. 1C).

**Figure 2:**
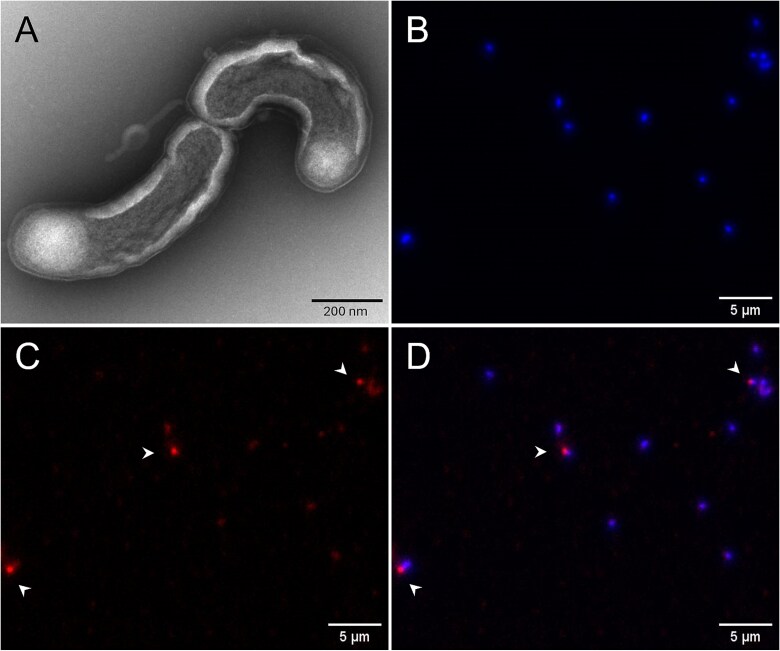
**Transmission electron and fluorescence microscopy of LSUCC0530 cells.** (A) TEM was performed on LSUCC0530 cells grown to late exponential phase in JW5. (B) LSUCC0530 cells were grown to early stationary phase in JW5, stained with Nile red (PHA stain, red) and DAPI (nucleic acid stain, blue), and imaged via epifluorescence microscopy. (B) DAPI channel; (C) TRITC channel; (D) merged image. Arrowheads indicate cells with Nile red-positive inclusions.

### “*Ca.* F. ubiquis” cells make polar, Nile red-staining inclusion bodies

As we had previously noted the presence of PHA production genes in the LSUCC0530 genome [38], the ability of LSUCC0530 to multiply in the absence of supplemental carbon raised the question of whether this organism could use stored carbon during starvation periods. In evaluating the morphology of LSUCC0530 cells via transmission electron microscopy (TEM), we found that many of the cells contained a single polar, ground glass-like inclusion body (Fig. 2A). With the notion that these inclusion bodies might be carbon storage granules, we stained LSUCC0530 cells with Nile red, a lipophilic dye that produces high-intensity fluorescence when bound to PHA [50]. Imaging via epifluorescence microscopy demonstrated that a fraction of LSUCC0530 cells contained a single, polar inclusion body that stained brightly with Nile red (Fig. 2B). These Nile red-staining inclusion bodies occurred in the same subcellular location as the inclusions we observed in the TEM images; when seen in dividing cells, the inclusions were always found at the pole opposite the site of cell division (Fig. 2A). Based on analysis of the size of the cellular inclusions in the TEM images, assuming the inclusions were composed of 100% PHB, we estimated that LSUCC0530 cells contained an average of 2.93 fg/cell (1.24–4.63 fg/cell).

### Genomes of a subset of SAR11 strains encode PHA enzyme homologs

The genomic potential for PHA production was previously described in genomic characterizations of LSUCC0530 and other SAR11 strains in both subclades IIIa and LD12 [7, 20]. The genomes of both LSUCC0530 and the SAR11 subclade IIIa strain LSUCC0261 contain loci predicted to encode homologs of the enzymes involved in both the production of PHA (PhaC, phasin family protein PhaP, PhaB, PhaA, and short chain enoyl-CoA hydratase PhaJ; encoded in LSUCC0530 by locus tags Ga0258990_1170–74) and its degradation (alpha/beta hydrolase PhaZ; encoded in LSUCC0530 by locus tag Ga0258990_1175). Unlike in other, well-studied organisms that produce PHA, many of which contain multiple copies of various *pha* genes [51, 52], the LSUCC0530 genome contains only a single copy of the core *phaA*, *phaB*, and *phaC* genes.

**Figure 3:**
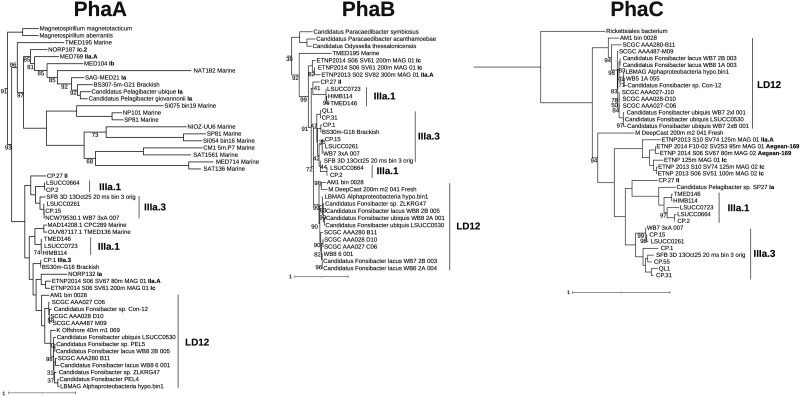
**Pha protein phylogeny in the SAR11 clade.** Maximum likelihood phylogenetic trees of Pha synthesis enzyme protein sequences annotated in SAR11 genomes. Node values indicate branch support bootstrap values (as a percentage; n = 1000) and are reported only for nodes with <100% support. Subclade assignments follow strain names; where subclade assignment is unavailable, water source type (brackish or marine) is provided. Expanded trees containing non-SAR11 homologs, accession numbers, and annotations are displayed in Fig. S1A–C.

Phylogenetic trees of the PhaA, PhaB, and PhaC protein sequences encoded in available SAR11 genomes demonstrated that the SAR11 homologs of these proteins formed a monophyletic group in each tree and were deeply divergent from those found in other species, including other *Alphaproteobacteria* (Fig. S1). Our protein search found homologs for the Pha proteins throughout the subclades of SAR11, but these proteins appear to be enriched in the IIIa and LD12 subclades. Pha proteins in these two subclades clustered into monophyletic groups (Fig. 3; Fig. S1), however, the Pha protein trees generally did not follow the branching order of the species trees for SAR11 (Fig. 3). For example, the SAR11 IIIa and LD12 PhaC clades were separated by sequences from SAR11 subclades Ia, Ic, II, and Aegean-169 sequences, even though IIIa and LD12 are taxonomically sister clades. This corroborates previous phylogenetic studies of *pha* genes and PhaC proteins that have illustrated the disconnect between 16S rRNA gene phylogeny and *pha* gene relatedness [21, 53] and suggests intra-SAR11 horizontal transfer of *pha* genes [54].

### Heterologous expression of *phaCBA* from LSUCC0530 causes production of Nile red-positive inclusion bodies

**Figure 4:**
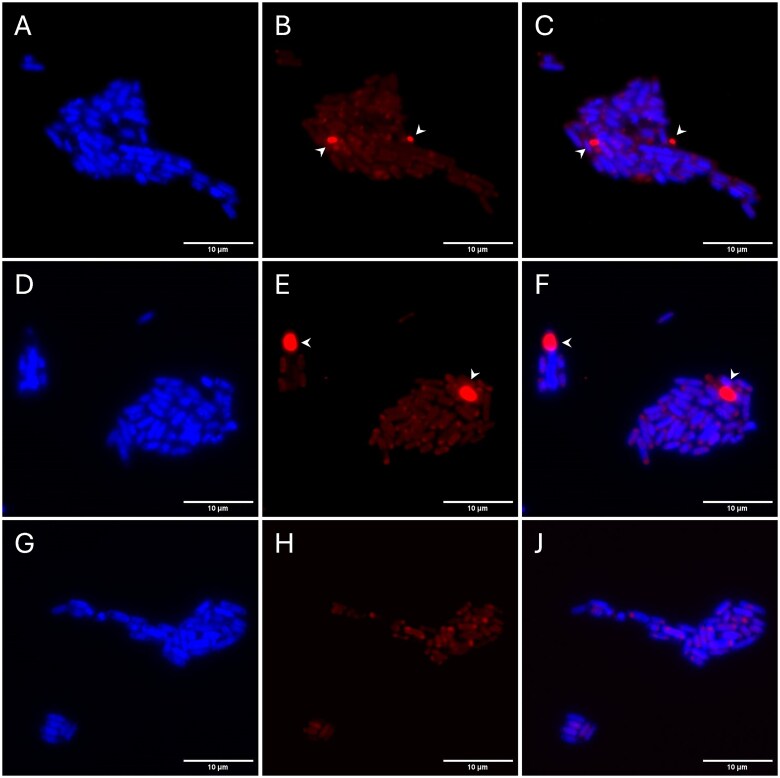
**Fluorescence microscopy of *E. coli* carrying heterologous *PHA* constructs.**  *E. coli* (A–C) expressing *phaCBA_FU_*, (D–F) expressing *phaCAB_CN_*, or (G–J) carrying empty vector was stained with Nile red (PHA stain, red) and DAPI (nucleic acid stain, blue) and imaged via epifluorescence microscopy. (A, D, and G) DAPI channel; middle, (E and H) TRITC channel; (C, F, and J) merged images. Arrowheads indicate cells with Nile red-positive inclusions.

To determine whether the LSUCC0530 *pha* locus is involved in making the presumptive carbon storage inclusion bodies observed in Fig. 2, we heterologously expressed the LSUCC0530 homologs of the core PHA production genes *phaC*, *phaB*, and *phaA* (*phaCBA_FU_*) in *E. coli*. We performed epifluorescence microscopy on *E. coli* complemented with empty vector, vector expressing *phaCBA_FU_*, or vector expressing *phaC1AB1* from *C. necator* H16 (*phaCAB_CN_*, positive control). We observed inclusion bodies that stained with Nile red in *E. coli* strains expressing either *phaCBA_FU_* or *phaCAB_CN_* (Fig. 4A and Fig. 4B); no inclusions were seen in *E. coli* cells carrying empty vector (Fig. 4C).

### LSUCC0530 cells produce PHB

**Figure 5:**
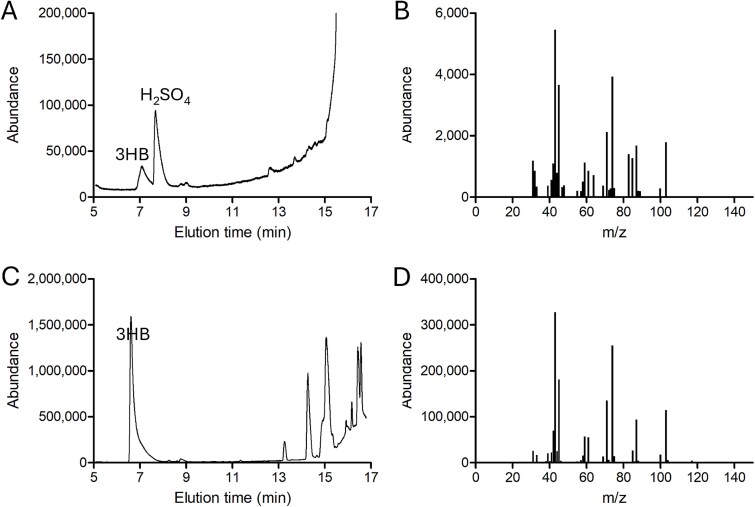
**GC/MS analysis of LSUCC0530 culture lipid extracts.** GC/MS was performed on lipid extracts from cultures of (A and B) LSUCC0530 grown into early stationary phase on CCM5PK and (C and D) *C. necator* grown into stationary phase on nutrient broth with gluconate. A and C, GC chromatograms; peaks are labeled with the highest-confidence match of the corresponding MS spectrum to the NIST database. B and D, MS spectra for the peaks labeled 3-hydroxybutyric acid (3HB). Note the higher scale for abundance in (C and D). H_2_SO_4_ is a reagent used during lipid extraction.

To confirm that the inclusion bodies observed in LSUCC0530 cells (Fig. 2) contained PHA, and to identify the presumptive PHA monomer(s), we conducted gas chromatography/mass spectrometry (GC/MS) on lipid extracts of LSUCC0530 cultures. The sole PHA derivative detected in the LSUCC0530 cells was 3-hydroxybuytyric acid (3HB), the monomer unit of PHB (Fig. 5A). We also observed 3HB production in a *C. necator* culture grown in nutrient broth with gluconate, serving as a positive control, with a similar GC retention time to the 3HB found in LSUCC0530 (Fig. 5B). No PHA monomer compounds were detected in extracts of a CCM5PK medium-only blank (Fig. S2). The PHB content averaged 1.21 fg/cell (0.087–3.39 fg/cell), which aligns with our estimate of cellular PHB content based on image analysis.

### Increasing nitrogen and phosphate concentrations does not affect PHA production in LSUCC0530

**Figure 6:**
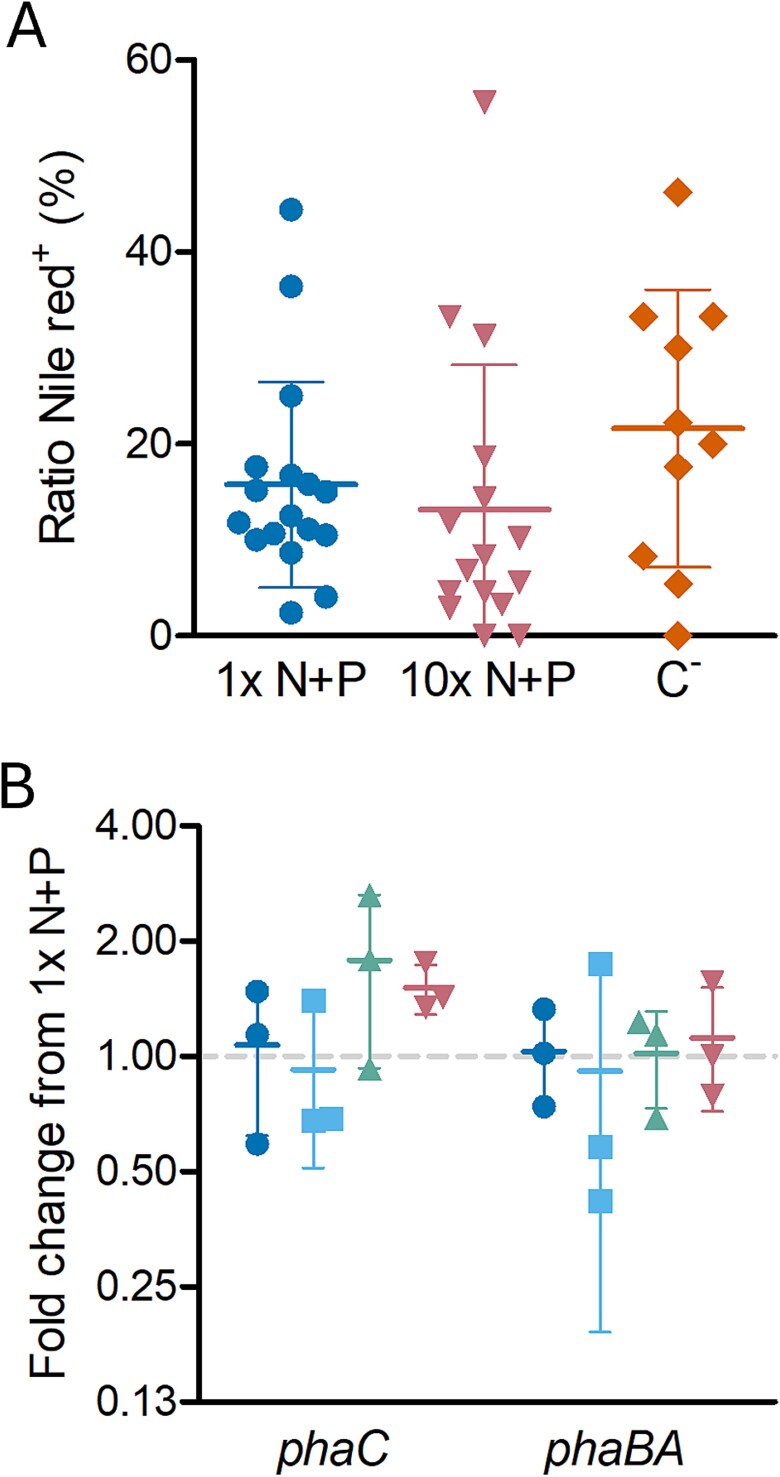
**Microscopic and transcriptional analysis of nitrogen and phosphorus influence on PHA production in LSUCC0530.** (A) The percentage of cells containing PHA inclusion bodies was counted for LSUCC0530 cultures grown to early stationary phase in JW5 with 1x ammonium and phosphate (circles), 10x ammonium and phosphate (inverted triangles), or without supplemental carbon (diamonds) and then stained with Nile red and DAPI and imaged via epifluorescence microscopy. Each point represents one biological replicate; five fields of view were analyzed for each replicate. Error bars represent ±1 standard deviation. (B) Expression of *phaC* and *phaBA* was analyzed via RT-qPCR for cultures of LSUCC0530 grown to early stationary phase in JW5 with 1x ammonium and phosphate (circles), 10x ammonium (squares), 10x phosphate (triangles), or 10x ammonium and phosphate (inverted triangles). Each point represents one biological replicate; values displayed are the fold change from that of the average of 1x JW5. Error bars represent ±1 standard deviation.

Nitrogen and phosphorus limitation commonly induce PHA production in many bacteria [33, 34]. To determine whether production of the PHA inclusions in LSUCC0530 is induced by nitrogen and/or phosphorous limitation, we quantified PHA inclusions via Nile red staining and epifluorescence imaging in LSUCC0530 cultures grown in JW5 with either the standard concentrations of ammonium and phosphate (5 μM and 6.5 μM, respectively) or 10x the concentrations of both compounds. We found no difference in the fraction of LSUCC0530 cells containing PHA inclusions between the two growth conditions (Fig. 6A). Additionally, we wanted to know whether LSUCC0530 cells grown in a medium lacking a supplemental carbon source would consume the stored PHA and, therefore, produce fewer progeny containing PHA inclusions. There was no difference in the fraction of LSUCC0530 cells containing PHA inclusions regardless of the presence or absence of supplemental carbon in the growth medium (Fig. 6A). Regardless of the growth condition, we found a high variance in the percentage of cells considered positive for PHA inclusions, from zero or near zero to greater than 40% (Fig. 6A). This suggests considerable subpopulation heterogeneity that could indicate a wide range in growth phenotypes depending on how the cells use PHB.

We also investigated whether nitrogen and/or phosphorous concentrations influence expression of PHA production genes using reverse transcription-quantitative PCR (RT-qPCR) on LSUCC0530 grown in JW5 with 1x or 10x ammonium and/or phosphate. We observed no difference in the expression levels of *phaC* or *phaBA* in LSUCC0530 grown in any of the tested media (Fig. 6B), suggesting that either PHB production was constitutive or that expression is responsive to different stimuli than for other known PHB-producing taxa.

## Discussion

Though the genomic potential for PHA production by LSUCC0530 and other SAR11 strains had previously been identified [7, 19], the activity of *pha* homolog genes had not been studied in any SAR11 strain before this work. In this study, we determined that the *pha* synthesis genes encoded in the LD12 strain LSUCC0530 are functional and that LSUCC0530 produces PHB irrespective of carbon, nitrogen, or phosphate concentration. Without tools available to modify the genomes of SAR11 bacteria, we were unable to inactivate the *pha* locus and investigate whether PHA production contributes to survival of LSUCC0530 under starvation conditions (Fig. 1A). Our estimate of cellular PHB concentration at a maximum of ~3 fg/cell, combined with the total carbon content of the similarly sized HTCC1062 at ~6.5 fg/cell [55], meant that PHB alone could not be responsible for the continued growth of LSUCC0530 without added carbon. As the LSUCC0530 genome is not predicted to encode the enzymes necessary for CO_2_ fixation, the full picture of how LSUCC0530 is able to continue to grow in carbon-depleted media remains unclear. We cannot exclude the possibility that LSUCC0530 is able to use one or more of the vitamins in our media as a carbon source, though we think it unlikely; flux-balance analysis would be required to determine if this is the case. However, we think it is possible that some of this growth is achieved by cells utilizing carbon stored as PHB. We have previously reported a similar phenotype in the OM252 *Gammaproteobacteria* representative strain LSUCC0096 [56]. Although strain LSUCC0096 has genes for autotrophy, it also has putative *pha* genes and could grow for multiple transfers without an added electron donor [56], implicating PHA storage in energy generation and possibly as a carbon supply.

The potential utilization of stored carbon by LSUCC0530 to support survival under starvation conditions may complement the function provided by proteorhodopsin, another survival mechanism widely possessed by bacteria throughout the *Pelagibacterales* [7, 38, 57, 58]. Proteorhodopsin absorbs light and pumps protons, serving to maintain membrane potential [57, 59]. In the subclade Ia strain HTCC1062, proteorhodopsin prevents oxidation of biomass during periods of starvation [58]. Given the ability of LSUCC0530 to continue growing under starvation conditions, PHA storage capability may provide LD12, subclade IIIa, and other PHA-producing SAR11 strains with a fitness advantage during periods of starvation, as PHA oxidation may provide energy as well as serve as a carbon source. The relative contribution of proteorhodopsin to the energy needs of PHA-oxidizing SAR11 cells deserves further study to determine whether and how these metabolisms are used in conjunction.

Unlike for many other organisms [33, 34], increased nitrogen and phosphorous concentrations do not appear to repress PHA accumulation in LSUCC0530 (Fig. 6). It is possible that PHA production in SAR11 bacteria is regulated via some other mechanism that we did not investigate. Indeed, as SAR11 are oligotrophic organisms, they are already well-adapted to the canonical nitrogen and phosphorous limitation that typically promote PHA production in other organisms [4]. Less commonly observed PHA regulatory mechanisms include oxygen restriction [60], iron limitation [61], and quorum signaling [62]; however, our cultures were grown aerobically with excess iron, and no evidence of quorum signaling in SAR11 bacteria has been discovered as yet [63]. Transcriptional regulators shown to affect *pha* gene expression and PHA production in other bacteria include the alternative sigma factors RpoN [64] and RpoS [65], though there are no genes encoding homologs of those sigma factors annotated in the LSUCC0530 genome. Currently there are no obvious potential mechanisms for regulating PHA production in LSUCC0530, making it likely that its *pha* synthesis genes are constitutively expressed.

A possible explanation for the apparent lack of PHA synthesis regulation in LSUCC0530 may be related to the slow growth phenotype of SAR11 bacteria. The highly streamlined genomes of SAR11 bacteria contain a single copy of the 5S, 16S, and 23S rRNA genes [9], and SAR11 cells have low ratios of rRNA/rDNA, both of which are linked to slow growth rates [66, 67]. However, SAR11 bacteria are at least as metabolically active as other prokaryotes [68]. It may be that the growth-limiting factor in SAR11 cells is the translation machinery, causing biomass production to lag behind catabolism and producing excess intracellular NADH and ATP—the very conditions that cause cells to shunt acetyl-CoA away from the TCA cycle and into PHA production [35, 69]. Thus, PHA production may be constitutive in those SAR11 strains whose genomes contain complete *pha* loci simply due to their particular physiology resulting from streamlined genomes. In these bacteria, it is possible that the major factor affecting the presence of PHA in a cell is the rate of PHA utilization, rather than synthesis.

The ratio of LSUCC0530 cells containing PHA inclusions was similar regardless of whether the bacteria were grown in media with or without a supplemental carbon source; in fact, the average proportion of PHA-containing cells was slightly higher in the carbon-deplete medium, though this was not statistically significant (Mann–Whitney t-test *P* = .27) (Fig. 6A). It is theoretically possible that the depolymerase PhaZ encoded in the LSUCC0530 genome is nonfunctional, as we did not test this hypothesis. However, we think it more likely that PhaZ is functional and is used to depolymerize PHA during periods of carbon limitation. We speculate that those cells that have used up their PHA stores are more likely to have died off and no longer be detectable via microscopy, leaving those still with available PHA to continue growing. The carbon-starved cultures used for microscopy grew to lower average densities (4.8x10^6^ ± 3.9x10^6^ cells/ml) than the carbon-replete cultures (1.2x10^7^ ± 6.5x10^6^ cells/ml), so although the ratio of Nile red-positive cells in the carbon-starved cultures was equal to or higher than that in the carbon-replete cultures, the total number of live cells was lower. It may be worthwhile in a future study to track both the ratio of PHA-containing LSUCC0530 cells in carbon-starved cultures and their PHB concentrations throughout a growth curve into death phase as well as over multiple transfers until cell division finally ends, to see if these values increase and then eventually decrease over time.

The wide range of LSUCC0530 cells containing visible PHA granules under the conditions we evaluated needs to be reconciled with our hypothesis of constitutive PHB synthesis. One possible explanation for this discrepancy is that in some cells, there is simply not enough accumulated PHA for detection. It was difficult to visualize subcellular structures in these ultramicrobacteria [2] using fluorescence microscopy, and it may be that smaller amounts of PHB in these cells are indistinguishable from background Nile red staining of the cell membrane. Additionally, the relative amount of PHB in each cell may reflect different amounts of utilization stemming from phenotypic heterogeneity among individual cells. If PHB production is constitutive and the amount found in a cell is due to the relative rate of consumption vs. production, then the amount of PHB may reflect higher or lower rates of a cell’s metabolic activity. For example, by modeling the viability of LD12 in natural populations, we estimated that over half of LD12 cells may be dormant at any one time [70]. If similar levels of quiescence occur in culture, that may partially explain the large variance in cellular PHB levels.

Genes encoding PHA enzyme homologs are found across all the major SAR11 subclades (Fig. 3, Fig. S1). These genes have previously been reported in the brackish and freshwater SAR11 subclades II, IIIa, and LD12 [7, 19, 20]; we have now also identified the presence of these genes within marine-dwelling SAR11 subclade I and Aegean-169. Though the potential for PHA production can be found across the entire SAR11 clade, only a subset of available SAR11 genomes encode homologs of Pha enzymes. The *pha* genes generally grouped by subclade (Fig. 3), although the subclade branching order was different from that of species trees generated with whole genomes [7]. Furthermore, *pha* genes were much more common in the fresh and brackish-water subclades LD12 and IIIa compared to other SAR11 subclades. Coupling these results with the observation that SAR11 Pha proteins are deeply divergent from those found in other *Alphaproteobacteria* suggests that the genes enabling PHA production were inherited by a common ancestor of the IIIa/LD12 lineages and then horizontally transferred very occasionally to other lineages within SAR11.

Pressing questions remain regarding the heterogeneity of PHA accumulation in LSUCC0530 cells under all tested conditions in vitro, and whether PHA granules would be seen in similar fractions of LSUCC0530 cells taken from freshwater environments undergoing either eutrophication or oligotrophication. It would also be worthwhile to explore whether the putative *pha* homologs present in cultivated SAR11 strains from the marine- and brackish-dwelling subclades are active and if these strains can continue propagating in the absence of a carbon source, as with the freshwater-evolved LSUCC0530. Further work should also investigate the possibility that other likely PHA-producing SAR11 strains have alternative regulatory mechanisms governing PHA production, and if so whether these mechanisms also exist in LSUCC0530.

## Supplementary Material

Supplementary_material_revised_wraf087

## Data Availability

The raw datasets and images generated in the work presented here are available for download at 10.6084/m9.figshare.28691048. Strain LSUCC0530 has been submitted to the American Type Culture Collection and is undergoing certification. Strain HTCC1062 was generously provided by Dr. Stephen Giovannoni and is available from his laboratory upon request. Transformed *E. coli* strains are also available upon request.

## References

[1] Bahr M, Hobbie JE, Sogin ML. Bacterial diversity in an arctic lake: a freshwater SAR11 cluster. *Aquat Microb Ecol* 1996;11:271–7. Available from: https://www.int-res.com/abstracts/ame/v11/n3/p271-277/. 10.3354/ame011271

[2] Rappé MS, Connon SA, Vergin KL et al. Cultivation of the ubiquitous SAR11 marine bacterioplankton clade. *Nature* 2002;418:630–3. 10.1038/nature00917 12167859

[3] Morris RM, Rappé MS, Connon SA et al. SAR11 clade dominates ocean surface bacterioplankton communities. *Nature* 2002;420:806–10. 10.1038/nature01240 12490947

[4] Giovannoni SJ . SAR11 bacteria: the most abundant plankton in the oceans. *Annu Rev Mar Sci* 2017;9:231–55. 10.1146/annurev-marine-010814-015934 27687974

[5] Mary I, Heywood JL, Fuchs BM et al. SAR11 dominance among metabolically active low nucleic acid bacterioplankton in surface waters along an Atlantic meridional transect. *Aquat Microb Ecol* 2006;45:107–13. Available from: https://www.int-res.com/abstracts/ame/v45/n2/p107-113/. 10.3354/ame045107

[6] Laghdass M, Catala P, Caparros J et al. High contribution of SAR11 to microbial activity in the north West Mediterranean Sea. *Microb Ecol* 2012;63:324–33. 10.1007/s00248-011-9915-7 21887519

[7] Lanclos VC, Rasmussen AN, Kojima CY et al. Ecophysiology and genomics of the brackish water adapted SAR11 subclade IIIa. *ISME J.* 2023;17:620–9. 10.1038/s41396-023-01376-2 36739346 PMC10030771

[8] Zwart G, Crump BC, van Agterveld MPK et al. Typical freshwater bacteria: an analysis of available 16S rRNA gene sequences from plankton of lakes and rivers. *Aquat Microb Ecol* 2002;28:141–55. Available from: https://www.int-res.com/abstracts/ame/v28/n2/p141-155/. 10.3354/ame028141

[9] Grote J, Thrash JC, Huggett MJ et al. Streamlining and Core genome conservation among highly divergent members of the SAR11 clade. *MBio* 2012;3:e00252–12. 10.1128/mbio.00252-12 22991429 PMC3448164

[10] Salcher MM, Pernthaler J, Posch T. Seasonal bloom dynamics and ecophysiology of the freshwater sister clade of SAR11 bacteria ‘that rule the waves’ (LD12). *ISME J.* 2011;5:1242–52. 10.1038/ismej.2011.8 21412347 PMC3146277

[11] Heinrich F, Eiler A, Bertilsson S. Seasonality and environmental control of freshwater SAR11 (LD12) in a temperate Lake (Lake Erken, Sweden). *Aquat Microb Ecol* 2013;70:33–44. 10.3354/ame01637

[12] Giovannoni SJ, Tripp HJ, Givan S et al. Genome streamlining in a cosmopolitan oceanic bacterium. *Science* 2005;309:1242–5. 10.1126/science.1114057 16109880

[13] Noell SE, Hellweger FL, Temperton B et al. A reduction of transcriptional regulation in aquatic oligotrophic microorganisms enhances fitness in nutrient-poor environments. *Microbiol Mol Biol Rev* 2023;87:e0012422–2. 10.1128/mmbr.00124-22 36995249 PMC10304753

[14] Eiler A, Mondav R, Sinclair L et al. Tuning fresh: radiation through rewiring of central metabolism in streamlined bacteria. *ISME J.* 2016;10:1902–14. 10.1038/ismej.2015.260 26784354 PMC5029164

[15] Giovannoni SJ, Thrash JC, Temperton B. Implications of streamlining theory for microbial ecology. *ISME J* 2014;8:1553–65. Available from: https://academic.oup.com/ismej/article/8/8/1553/7582522. 10.1038/ismej.2014.60 24739623 PMC4817614

[16] Cottrell MT, Kirchman DL. Transcriptional control in marine Copiotrophic and oligotrophic bacteria with streamlined genomes. *Appl Environ Microbiol* 2016;82:6010–8. 10.1128/AEM.01299-16 27474718 PMC5038029

[17] Williams TJ, Joux F, Lauro FM et al. Physiology of marine oligotrophic Ultramicrobacteria. In: Horikoshi K, Antranikian G, Bull AT et al. (eds). Extremophiles Handbook. Tokyo: Springer Japan, 2011, 1179–99. 10.1007/978-4-431-53898-1_57

[18] Luo H, Csűros M, Hughes AL et al. Evolution of divergent life history strategies in marine Alphaproteobacteria. *MBio* 2013;4:e00373-13. 10.1128/mBio.00373-13 PMC3735120 23839216

[19] Campbell BJ, Lim SJ, Kirchman DL. Controls of SAR11 subclade abundance, diversity, and growth in two mid-Atlantic estuaries. *bioRxiv* 2022; 2022.05.04.490708. Available from: 10.1101/2022.05.04.490708

[20] Oh S, Zhang R, Wu QL et al. Evolution and adaptation of SAR11 and *Cyanobium* in a saline Tibetan lake. *Environ Microbiol Rep* 2016;8:595–604. 10.1111/1758-2229.12408 27084571

[21] Kalia VC, Lal S, Cheema S. Insight in to the phylogeny of polyhydroxyalkanoate biosynthesis: horizontal gene transfer. *Gene* 2007;389:19–26. 10.1016/j.gene.2006.09.010 17113245

[22] Stockdale H, Ribbons DW, Dawes EA. Occurrence of poly-β-Hydroxybutyrate in the Azotobacteriaceae. *J Bacteriol* 1968;95:1798–803. 10.1128/jb.95.5.1798-1803.1968 5650085 PMC252214

[23] Anderson AJ, Dawes EA. Occurrence, metabolism, metabolic role, and industrial uses of bacterial polyhydroxyalkanoates. *Microbiol Rev* 1990;54:450–72. 10.1128/mr.54.4.450-472.1990 2087222 PMC372789

[24] Mason-Jones K, Robinson SL, Veen Ciska GF et al. Microbial storage and its implications for soil ecology. *ISME J.* 2022;16:617–29. 10.1038/s41396-021-01110-w 34593996 PMC8857262

[25] Madison LL, Huisman GW. Metabolic engineering of poly(3-Hydroxyalkanoates): from DNA to plastic. *Microbiol Mol Biol Rev* 1999;63:21–53. 10.1128/mmbr.63.1.21-53.1999 10066830 PMC98956

[26] Peoples OP, Masamune S, Walsh CT et al. Biosynthetic thiolase from *Zoogloea ramigera*. III. Isolation and characterization of the structural gene. *J Biol Chem* 1987;262:97–102. 10.1016/S0021-9258(19)75893-1 2878929

[27] Peoples OP, Sinskey AJ. Poly-β-hydroxybutyrate (PHB) biosynthesis in *Alcaligenes eutrophus* H16: identification and characterization of the PHB polymerase gene (*phbC*). *J Biol Chem* 1989;264:15298–303. 10.1016/S0021-9258(19)84825-1 2670936

[28] Steinbüchel A, Valentin HE. Diversity of bacterial polyhydroxyalkanoic acids. *FEMS Microbiol Lett* 1995;128:219–28. 10.1111/j.1574-6968.1995.tb07528.x

[29] Zher Neoh S, Fey Chek M, Tiang Tan H et al. Polyhydroxyalkanoate synthase (PhaC): the key enzyme for biopolyester synthesis. *Curr Res Biotechnol* 2022;4:87–101. 10.1016/j.crbiot.2022.01.002

[30] Moradali MF, Rehm BHA. Bacterial biopolymers: from pathogenesis to advanced materials. *Nat Rev Microbiol* 2020;18:195–210. 10.1038/s41579-019-0313-3 31992873 PMC7223192

[31] Mitra R, Xu T, Chen GQ et al. An updated overview on the regulatory circuits of polyhydroxyalkanoates synthesis. *Microb Biotechnol* 2022;15:1446–70. 10.1111/1751-7915.13915 34473895 PMC9049629

[32] Steinbüchel A, Lütke-Eversloh T. Metabolic engineering and pathway construction for biotechnological production of relevant polyhydroxyalkanoates in microorganisms. *Biochem Eng J* 2003;16:81–96. 10.1016/S1369-703X(03)00036-6

[33] Schlegel HG, Gottschalk G, Von Bartha R. Formation and utilization of poly-β-Hydroxybutyric acid by Knallgas bacteria (*Hydrogenomonas*). *Nature* 1961;191:463–5. 10.1038/191463a0 13747776

[34] Ballard DGH, Holmes PA, Senior PJ. Formation of polymers of ß-Hydroxybutyric acid in bacterial cells and a comparison of the morphology of growth with the formation of polyethylene in the solid state. In: Fontanille M, Guyot A. (eds.), Recent Advances in Mechanistic and Synthetic Aspects of Polymerization. Dordrecht: Springer Netherlands, 1987, 293–314 10.1007/978-94-009-3989-9_22

[35] Senior PJ, Dawes EA. The regulation of poly-β-hydroxybutyrate metabolism in *Azotobacter beijerinckii*. *Biochem J* 1973;134:225–38. 10.1042/bj1340225 4723225 PMC1177803

[36] Oeding V, Schlegel HG. p-Ketothiolase from *Hydrogenomonas eutropha* H16 and its significance in the regulation of poly-p-hydroxybutyrate metabolism. *Biochem J* 1973;134:239–48. 10.1042/bj1340239 4198758 PMC1177804

[37] Miyake M, Kataoka K, Shirai M et al. Control of poly-beta-hydroxybutyrate synthase mediated by acetyl phosphate in cyanobacteria. *J Bacteriol* 1997;179:5009–13. 10.1128/jb.179.16.5009-5013.1997 9260940 PMC179356

[38] Henson MW, Lanclos VC, Faircloth BC et al. Cultivation and genomics of the first freshwater SAR11 (LD12) isolate. *ISME J.* 2018;12:1846–60. 10.1038/s41396-018-0092-2 29599519 PMC6018831

[39] Carini P, Steindler L, Beszteri S et al. Nutrient requirements for growth of the extreme oligotroph ‘*Candidatus* Pelagibacter ubique’ HTCC1062 on a defined medium. *ISME J* 2013;7:592–602. 10.1038/ismej.2012.122 23096402 PMC3578571

[40] Schindelin J, Arganda-Carreras I, Frise E et al. Fiji: an open-source platform for biological-image analysis. *Nat Methods* 2012;9:676–82. 10.1038/nmeth.2019 22743772 PMC3855844

[41] Barham PJ, Keller A, Otun EL et al. Crystallization and morphology of a bacterial thermoplastic: poly-3-hydroxybutyrate. *J Mater Sci* 1984;19:2781–94. 10.1007/BF01026954

[42] Gervaise AL, Arur S. Spatial and temporal analysis of active ERK in the *C. Elegans* germline. *J Vis Exp* 2016:e54901. 10.3791/54901 PMC5226324 27929466

[43] Altschul SF, Madden TL, Schäffer AA et al. Gapped BLAST and PSI-BLAST: a new generation of protein database search programs. *Nucleic Acids Res* 1997;25:3389–402. 10.1093/nar/25.17.3389 9254694 PMC146917

[44] Edgar RC . MUSCLE: multiple sequence alignment with high accuracy and high throughput. *Nucleic Acids Res* 2004;32:1792–7. 10.1093/nar/gkh340 15034147 PMC390337

[45] Capella-Gutiérrez S, Silla-Martínez JM, Gabaldón T. trimAl: a tool for automated alignment trimming in large-scale phylogenetic analyses. *Bioinformatics* 2009;25:1972–3. 10.1093/bioinformatics/btp348 19505945 PMC2712344

[46] Minh BQ, Schmidt HA, Chernomor O et al. IQ-TREE 2: new models and efficient methods for phylogenetic inference in the genomic era. *Mol Biol Evol* 2020;37:1530–4. 10.1093/molbev/msaa015 32011700 PMC7182206

[47] Letunic I, Bork P. Interactive tree of life (iTOL) v5: an online tool for phylogenetic tree display and annotation. *Nucleic Acids Res* 2021;49:W293–6. 10.1093/nar/gkab301 33885785 PMC8265157

[48] Kovach ME, Elzer PH, Steven Hill D et al. Four new derivatives of the broad-host-range cloning vector pBBR1MCS, carrying different antibiotic-resistance cassettes. *Gene* 1995;166:175–6. 10.1016/0378-1119(95)00584-1 8529885

[49] Juengert J, Bresan S, Jendrossek D. Determination of Polyhydroxybutyrate (PHB) content in *Ralstonia eutropha* using gas chromatography and Nile red staining. *Bio Protoc* 2018;8:1–15. 10.21769/BioProtoc.2748 PMC8203877 34179275

[50] Degelau A, Scheper T, Bailey JE et al. Fluorometric measurement of poly-β hydroxybutyrate in *Alcaligenes eutrophus* by flow cytometry and spectrofluorometry. *Appl Microbiol Biotechnol* 1995;42:653–7. 10.1007/BF00171939

[51] Timm A, Steinbüchel A. Cloning and molecular analysis of the poly(3-hydroxyalkanoic acid) gene locus of *Pseudomonas aeruginosa* PAO1. *Eur J Biochem* 1992;209:15–30. 10.1111/j.1432-1033.1992.tb17256.x 1396693

[52] Pohlmann A, Fricke WF, Reinecke F et al. Genome sequence of the bioplastic-producing “Knallgas” bacterium *Ralstonia eutropha* H16. *Nat Biotechnol* 2006;24:1257–62. 10.1038/nbt1244 16964242

[53] Kutralam-Muniasamy G, Corona-Hernandez J, Narayanasamy RK et al. Phylogenetic diversification and developmental implications of poly-(R)-3-hydroxyalkanoate gene cluster assembly in prokaryotes. *FEMS Microbiol Lett* 2017;364:fnx135. 10.1093/femsle/fnx135 28655209

[54] Zaremba-Niedzwiedzka K, Viklund J, Zhao W et al. Single-cell genomics reveal low recombination frequencies in freshwater bacteria of the SAR11 clade. *Genome Biol* 2013;14:R130. 10.1186/gb-2013-14-11-r130 24286338 PMC4053759

[55] White AE, Giovannoni SJ, Zhao Y et al. Elemental content and stoichiometry of SAR11 chemoheterotrophic marine bacteria. *Limnol Oceanogr Lett* 2019;4:44–51. 10.1002/lol2.10103

[56] Savoie ER, Lanclos VC, Henson MW et al. Ecophysiology of the cosmopolitan OM252 Bacterioplankton (Gammaproteobacteria). *mSystems* 2021;6:e00276–21. 10.1128/mSystems.00276-21 34184914 PMC8269220

[57] Giovannoni SJ, Bibbs L, Cho JC et al. Proteorhodopsin in the ubiquitous marine bacterium SAR11. *Nature* 2005;438:82–5. 10.1038/nature04032 16267553

[58] Steindler L, Schwalbach MS, Smith DP et al. Energy starved *Candidatus* Pelagibacter Ubique substitutes light-mediated ATP production for endogenous carbon respiration. *PLoS One* 2011;6:e19725. 10.1371/journal.pone.0019725 21573025 PMC3090418

[59] Béjà O, Aravind L, Koonin EV et al. Bacterial rhodopsin: evidence for a new type of Phototrophy in the sea. *Science* 2000;289:1902–6. 10.1126/science.289.5486.1902 10988064

[60] Tribelli PM, Méndez BS, López NI. Oxygen-sensitive global regulator, Anr, is involved in the biosynthesis of poly(3-Hydroxybutyrate) in *pseudomonas extremaustralis*. *J Mol Microbiol Biotechnol* 2010;19:180–8. 10.1159/000320261 21042031

[61] Muriel-Millán LF, Castellanos M, Hernandez-Eligio JA et al. Posttranscriptional regulation of PhbR, the transcriptional activator of polyhydroxybutyrate synthesis, by iron and the sRNA ArrF in *Azotobacter vinelandii*. *Appl Microbiol Biotechnol* 2014;98:2173–82. 10.1007/s00253-013-5407-7 24305738

[62] Mohanan N, Gislason A, Sharma PK et al. Quorum sensing and the anaerobic regulator (ANR) control polyhydroxyalkanoate (PHA) production in *pseudomonas chlororaphis* PA23. *FEMS Microbiol Lett* 2019;366:fnz223. 10.1093/femsle/fnz223 31688920

[63] Lami R. Quorum sensing in marine biofilms and environments. In: Giuseppina Tommonaro (ed). Quorum Sensing. London, United Kingdom: Elsevier, 2019, 55–96. 10.1016/B978-0-12-814905-8.00003-4

[64] Hoffmann N, Rehm BHA. Nitrogen-dependent regulation of medium-chain length polyhydroxyalkanoate biosynthesis genes in pseudomonads. *Biotechnol Lett* 2005;27:279–82. 10.1007/s10529-004-8353-8 15742151

[65] Raiger-Iustman LJ, Ruiz JA. The alternative sigma factor, σS, affects polyhydroxyalkanoate metabolism in *pseudomonas putida*. *FEMS Microbiol Lett* 2008;284:218–24. 10.1111/j.1574-6968.2008.01203.x 18498401

[66] Campbell BJ, Yu L, Heidelberg JF et al. Activity of abundant and rare bacteria in a coastal ocean. *Proc Nat Acad Sci* 2011;108:12776–81. 10.1073/pnas.1101405108 21768380 PMC3150899

[67] Lankiewicz TS, Cottrell MT, Kirchman DL. Growth rates and rRNA content of four marine bacteria in pure cultures and in the Delaware estuary. *ISME J.* 2016;10:823–32. 10.1038/ismej.2015.156 26394004 PMC4796920

[68] Malmstrom RR, Kiene RP, Cottrell MT et al. Contribution of SAR11 bacteria to dissolved Dimethylsulfoniopropionate and amino acid uptake in the North Atlantic Ocean. *Appl Environ Microbiol* 2004;70:4129–35. 10.1128/AEM.70.7.4129-4135.2004 15240292 PMC444831

[69] Lee IY, Kim MK, Chang HN et al. Regulation of poly-β-hydroxybutyrate biosynthesis by nicotinamide nucleotide in *Alcaligenes eutrophus*. *FEMS Microbiol Lett* 1995;131:35–9.

[70] Henson MW, Lanclos VC, Pitre DM et al. Expanding the diversity of Bacterioplankton isolates and Modeling isolation efficacy with large-scale dilution-to-extinction cultivation. *Appl Environ Microbiol* 2020;86:e00943–20. 10.1128/AEM.00943-20 32561583 PMC7440811

