# Supplementary material for: Polyhydroxybutyrate production by freshwater SAR11 (LD12)
Source: ISME J. 2025 Apr 30;19(1):wraf087. doi: 10.1093/ismejo/wraf087 (PMC12113158; doi:10.1093/ismejo/wraf087)
Supplement: Supplementary_material_revised_wraf087 [file supplementary_material_revised_wraf087.pdf]

Fig S1 A

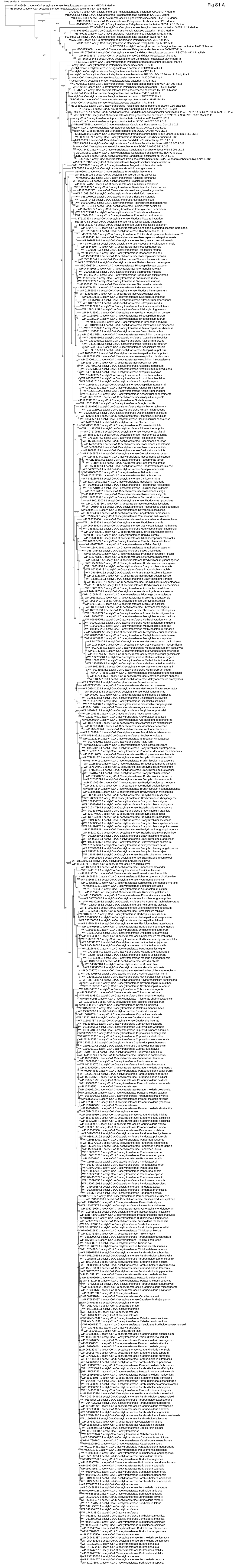

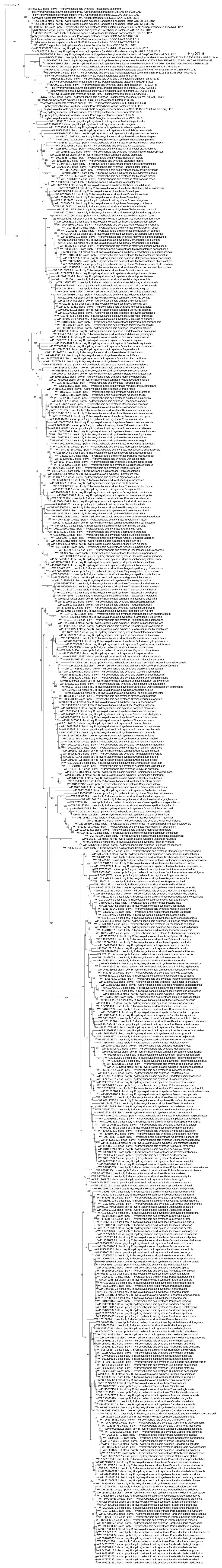

Fig S1 B

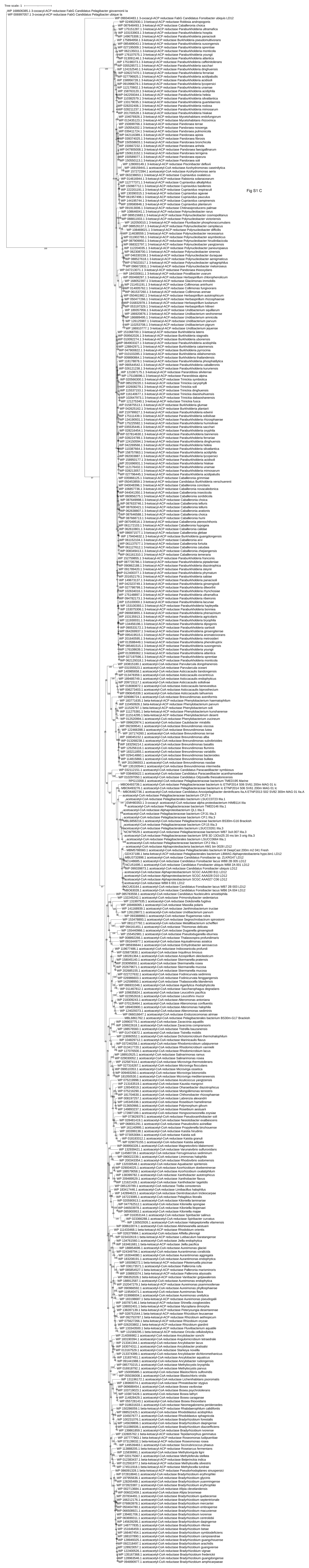
Tree scale: 1
Fig S1 C

Fig S1. Expanded Pha protein phylogeny. Maximum likelihood phylogenetic trees of Pha synthesis enzyme protein sequences annotated in bacterial genomes. A) PhaA homologs; B) PhaB homologs; C) PhaC homologs. Node values indicate branch support bootstrap values (max=100). SAR11 subclade assignments follow strain names; where subclade assignment is unavailable, water source type (fresh, brackish, or marine) is provided. Strain names are replaced by species names for RefSeq proteins. Proteins designated FabG (B) are annotated as 3-oxoacyl-[acyl-carrier-protein] reductase, which may replace PhaB during PHA synthesis.

Figure S2

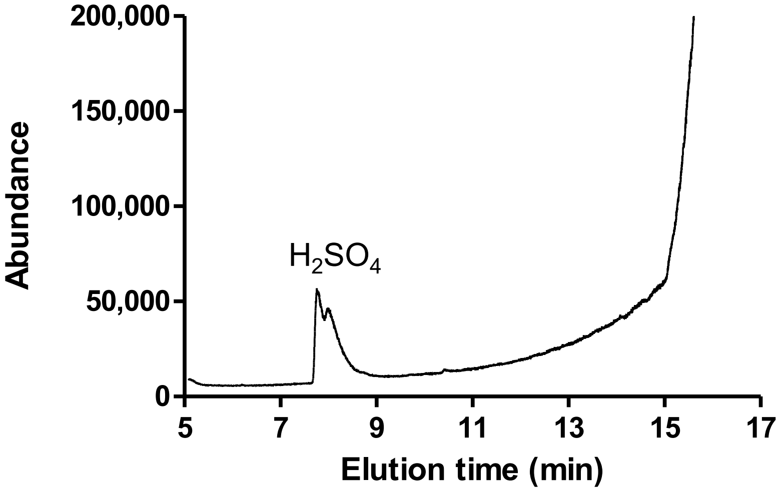

Fig S2. GC/MS analysis of medium blank lipid extract. GC/MS was performed on a lipid extract from a CCM5PK medium blank. GC chromatogram is shown; the peak is labeled with the highest confidence match of the corresponding MS spectrum to the NIST database. H_2SO_4 is a reagent used during lipid extraction.

Figure S3

Transfer	Max yield (cells/mL)	Generations	Minimum Doubling Time (d)	Minimum Doubling Time 1 SD
T1	3837657	10.1	2.30	4.24
T2	3230394	9.2	2.22	0.06
T3	3951815	8.6	2.04	0.38
T4	2361000	8.4	2.66	0.14
T5	3751333	10.1	2.21	0.35
T6	3426000	9.2	2.13	0.18
T7	4927333	9.2	2.23	0.17
T8	903333	7.2	2.22	*a*
T9	1992667	9.6	2.29	13.72

[a] Uncalculatable standard deviation

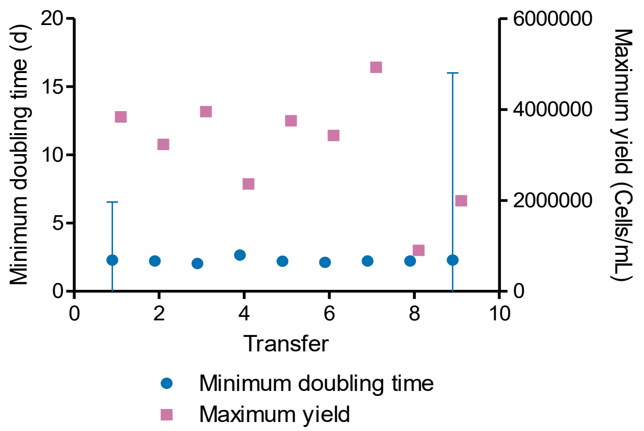

Fig S3. Growth rate, yield, and generation calculations for carbon-limited growth curves. Maximum growth yield, number of generations, and minimum doubling times were calculated for each LSUCC0530 carbon-limited culture depicted in Fig 1B. Circles, minimum doubling time; squares, maximum growth yield.

Table S1. Media used in this study

JW5

Component	Concentration
Salts and buffer	
NaCl	16.9 mM
KCl	416 µM
$NaHCO_3$	10 mM
Na_2SO_4	1.25 mm
NaBr	33 µM
H_3BO_3	16.2 µM
$SrCl_2$	3.75 µM
NaF	3.1 µM
KH_2PO_4	6.5 µM
$MgCl_2·6H_2O$	2.2 mM
$CaCl_2·2H_2O$	434 µM
Trace metals	
$FeSO_4·7H_2O$	101 nM
Nitrilotriacetic acid, disodium salt	345 nM
$MnCl_2·4H_2O$	9.1 nM
$ZnSO_4·H_2O$	1.11 nM
$CoCl_2$	500 pM
Na_2MoO_4	319 pM
Na_2SeO3	1 nM
$NiCl_2$	1 nM
Vitamins	
Thiamine	501 nM
Riboflavin	691 pM
Niacin	800 nM
Pantothenic acid	425 nM
Pyridoxine	500 nM
Biotin	4.01 nM
Folic acid	4.01 nM
Cobalamin	701 pM
Myo-inositol	500 nM
4-Aminobenzoic acid	60 nM
Carbon, sulfur, and nitrogen sources	
MEM Amino Acids (50x) Solution (Sigma-Aldrich)	0.001x
L-Glutamine	500 nM
Dextrose	500 nM
D-Ribose	500 nM
Sodium pyruvate	500 nM
Sodium citrate	500 nM
Oxaloacetic acid	500 nM
Sodium acetate	500 nM
Sodium succinate	500 nM
α-Ketoglutaric acid	500 nM
Urea	5 µM
Octanoic acid	500 nM
Decanoic acid	500 nM
Isobutyric acid	500 nM
Butyric acid	500 nM
Valeric acid	500 nM
$NaNO_3$	38 µM
$NaNO_2$	2 µM
NH_4Cl	5 µM

Carbon-Depleted JW5

Component	Concentration
Salts and buffer	
NaCl	16.9 mM
KCl	416 µM
$NaHCO_3$	10 mM
Na_2SO_4	1.25 mm
NaBr	33 µM
H_3BO_3	16.2 µM
$SrCl_2$	3.75 µM
NaF	3.1 µM
KH_2PO_4	6.5 µM
$MgCl_2·6H_2O$	2.2 mM
$CaCl_2·2H_2O$	434 µM
Trace metals	
$FeSO_4·7H_2O$	101 nM
Nitrilotriacetic acid, disodium salt	345 nM
$MnCl_2·4H_2O$	9.1 nM
$ZnSO_4·H_2O$	1.11 nM
$CoCl_2$	500 pM
Na_2MoO_4	319 pM
Na_2SeO3	1 nM
$NiCl_2$	1 nM
Vitamins	
Thiamine	501 nM
Riboflavin	691 pM
Niacin	800 nM
Pantothenic acid	425 nM
Pyridoxine	500 nM
Biotin	4.01 nM
Folic acid	4.01 nM
Cobalamin	701 pM
Myo-inositol	500 nM
4-Aminobenzoic acid	60 nM
Sulfur and nitrogen sources	
L-Methionine	10 µM
NH_4Cl	50 µM

CCM5PK

Component	Concentration
Salts and buffer	
NaCl	16.9 mM
KCl	417 µM
$NaHCO_3$	10 mM
$MgCl_2·6H_2O$	2.2 mM
$CaCl_2·2H_2O$	434 µM
KH_2PO_4	100 µM
Trace metals	
$FeSO_4·7H_2O$	101 nM
Nitrilotriacetic acid, disodium salt	345 nM
$MnCl_2·4H_2O$	9.1 nM
$ZnSO_4·H_2O$	1.11 nM
$CoCl_2$	500 pM
Na_2MoO_4	319 pM
Na_2SeO3	1 nM
$NiCl_2$	1 nM
Vitamins	
Thiamine	10.02 µM
Riboflavin	13.82 nM
Niacin	16 µM
Pantothenic acid	8.5 µM
Pyridoxine	10 µM
Biotin	80.2 nM
Folic acid	80.2 nM
Cobalamin	14.02 nM
Myo-inositol	10 µM
4-Aminobenzoic acid	1.2 µM
Carbon, sulfur, and nitrogen sources	
L-Methionine	20.1 µM
NH_4Cl	2 mM
Sodium pyruvate	999.6 µM
α-Ketoglutaric acid	691 µM

AMS1

Component	Concentration
Salts and buffer	
NaCl	481 mM
KCl	9 mM
$NaHCO_3$	6 mM
$MgCl_2·6H_2O$	27 mM
$CaCl_2·2H_2O$	10 mM
$MgSO_4·7H_2O$	2.8 mM
KH_2PO_4	50 µM
Trace metals	
$FeCl_3·6H_2O$	117 nM
$MnCl_2·4H_2O$	9 nM
$ZnSO_4·H_2O$	800 pM
$CoCl_2$	500 pM
Na_2MoO_4	300 pM
Na_2SeO_3	1 nM
$NiCl_2$	1 nM
Vitamins	
Thiamine	6 µM
Niacin	800 nM
Pantothenic acid	425 nM
Pyridoxine	500 nM
Biotin	4 nM
Folic Acid	4 nM
Cobalamin	700 pM
Myo-inositol	6 µM
4-Aminobenzoic acid	60 nM
Carbon, sulfur, and nitrogen sources	
L-Methionine	10 µM
L-Glycine	50 µM
$(NH_4)_2SO_4$	400 µM
Sodium pyruvate	50 µM
